# Optical studies and dielectric response of [DMA]_2_MCl_4_ (M = Zn and Co) and [DMA]_2_ZnBr_4_

**DOI:** 10.1039/d1ra03652a

**Published:** 2021-07-13

**Authors:** N. Mahfoudh, K. Karoui, A. BenRhaiem

**Affiliations:** Laboratory of Spectroscopic Characterization and Optic Materials, Faculty of Sciences, University of Sfax B. P. 1171 3000 Sfax Tunisia abdallahrhaiem@yahoo.fr

## Abstract

[DMA]_2_ZnCl_4_, [DMA]_2_CoCl_4_ and [DMA]_2_ZnBr_4_ crystallized in the monoclinic system, in the *P*2_1_/*n*, *P*2_1_/*n* and *P*2_1_/*c* space groups, respectively. The optical properties of [DMA]_2_MCl_4_ (M = Zn and Co) and [DMA]_2_ZnBr_4_ were studied using ultraviolet-visible (UV-Vis) spectroscopy in the range of 200–800 nm. The Tauc model was used to determine the band gap energy of each hybrid compound. The calculated values of the direct and indirect band gaps (*E*_gd_, *E*_gi_) for all samples were found to be in the range of 1.91 eV to 4.29 eV for [DMA]_2_ZnCl_4_, 4.76 eV to 5.34 eV for [DMA]_2_ZnBr_4_ and 1.77 eV to 3.84 eV for [DMA]_2_CoCl_4_. The Urbach energy (*E*_u_), extinction coefficient (*k*) and refractive index (*n*) of each compound was calculated. On the other hand, the dispersion of (*n*) is discussed in terms of the single oscillator Wemple–DiDomenico model. The single oscillator energy (*E*_0_), the dispersion energy (*E*_d_), and both the real *ε*_r_ and imaginary parts *ε*_i_ of the dielectric permittivity were estimated. The variation of optical conductivity with the incident photon energy has also been studied. We employed impedance spectroscopy to thoroughly investigate the dipolar dynamics in the prepared materials. The evolution of the dielectric loss, as a function of frequency, showed a distribution of relaxation times, which probably could be of a Maxwell–Wagner type interfacial polarization relaxation, possibly attributed to grain boundary effects or blocking at the contacts. In fact, the current work opens an efficient path to high quality organic–inorganic halide perovskites with good optical properties, which makes them suitable for application in nonlinear optoelectronic devices.

## Abstract 2

[DMA]_2_ZnCl_4_, [DMA]_2_CoCl_4_ and [DMA]_2_ZnBr_4_ crystallized in the monoclinic system, in the *P*2_1_/*n*, *P*2_1_/*n* and *P*2_1_/*c* space groups, respectively.

## Graphical abstract

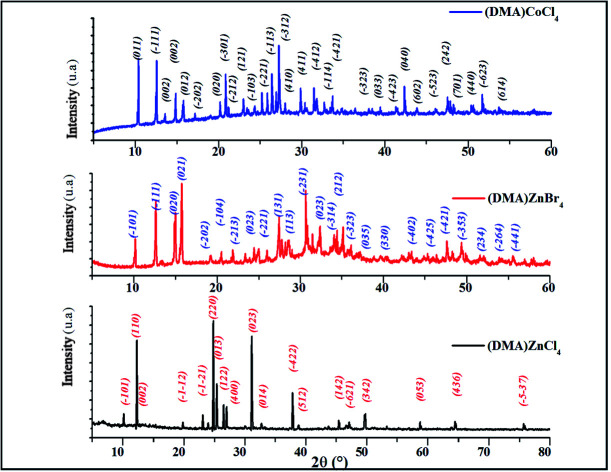

## 1. Introduction

[N(CH_3_)_2_H_2_]_2_ZnCl_4_, [N(CH_3_)_2_H_2_]_2_CoCl_4_ and [N(CH_3_)_2_H_2_]_2_ZnBr_4_ (here in after [DMA]_2_MeX_4_ (Me = Zn and Co and X = Cl and Br)) are members of the A_2_MeX_4_ crystal family, which have been extensively studied as typical materials showing various successive phase transitions when the temperature varies. On the other hand, the A_2_MeX_4_ type crystals are interesting because of their many physical properties related to the ferroelectric and commensurate or incommensurate phase transitions. Moreover, most of these materials show many physical properties related to structural phase transitions at low temperatures. Therefore, these fundamental properties make the crystal family suitable for several applications, such as temperature and humidity sensors, and memory effects that manifest themselves as temperature anomalies in their physical properties.^1–3^

Dimethylammonium tetrachlorozincate [DMA]_2_ZnCl_4_, dimethylammonium tetrachlorocolbatate [DMA]_2_CoCl_4_ and dimethylammonium tetrabromozincate [DMA]_2_ZnBr_4_ crystals have turned out to be very interesting members belonging to an A_2_MeX_4_ that undergoes different phase transitions at and below room temperature. In fact, at room temperature, [DMA]_2_ZnCl_4_ crystallizes in the monoclinic system with the *P*2_1_/*n* space group. Then, the refined parameters are: *a* = 13,297 Å, *b* = 8,620 Å, *c* = 11 494 Å and *β* = 89, 8°.^4,5^ However, the phase transition temperatures of [DMA]_2_ZnCl_4_ have not yet been correctly established. Therefore, an investigation of optical birefringence and piezoelectric coefficients revealed a range of phase transitions occurring at 217 K, 238 K, 250 K, 272 K, 295 K, and 310 K.^6^ Therefore, the temperatures of some phase transitions were determined through investigations of specific heat at 151 K, 240 K, 253.9 K, 272.2 K, 298.3 K, and 309.1 K.^7^ In addition, heat capacity measurements showed phase transitions at 201 K, 250 K, 272 K, and 310 K.^8^ Moreover, the occurrence of phase transitions at 444 K was confirmed by DSC measurements.^9^ Monoclinic crystals of [DMA]_2_CoCl_4_ are of the space group *P*2_1_/*n*. Unit cell dimensions are as follows: *a* 8.5313 Å, *b* = 11.4382 Å, *c* = 13.3070 Å and *β* = 90.038°.^10^ The transition temperatures of [DMA]_2_CoCl_4_ are *T*_1_ = 292 K, *T*_2_ = 319 K, *T*_3_ = 365 K, *T*_4_ = 374 K and *T*_5_ = 444.^11^ At 291 K, [DMA]_2_ZnBr_4_ crystallizes in the monoclinic system with the *P*2_1_/*c* space group and its unit cell parameters are *a* = 8.706 (6) Å, *b* = 11.956 (64) Å, *c* = 16.289 (95) Å, *β* = 121.84°, and *Z* = 4. The [DMA]_2_ZnBr_4_ crystal undergoes four phase transitions at *T*_1_ = 281 K, *T*_2_ = 340 K, *T*_3_ = 377 K, and *T*_4_ = 408 K.^12^

Theoretical and experimental studies of different crystals with dialkylammonium cation showed that the change of the hydrogen bond network considerably influences the lattice dynamics and manifests itself in an essential transformation of the crystalline structure. Therefore, due to the availability of H bonds, the above compounds are expected to be proton conductors. As a consequence, they are important because of the proton transport mechanisms in biophysical processes and their employment in numerous electrochemical devices, such as batteries, fuel cells, chemical sensors, electrochromic devices, and super capacitors.^13^

During the past years, optical studies have been performed on organic–inorganic nanocomposites. These studies have evolved towards different objectives, such as the development of materials with specific optical properties based on the properties of organic and inorganic chromophores. In fact, because of their several advantages for designing materials for optical applications (versatile and relatively facile chemistry, materials having good mechanical integrity and excellent optical quality), numerous hybrid organic–inorganic materials have been developed in the past few years. The chemical industry has tended to frequently use organometallic complexes because they use a minimum of energy by participating in chemical reactions in the transformation of natural elements. It should also be noted that organometallic complexes are known for their interesting luminescence properties, which have recently played an important role, particularly as phosphorescent components for light-emitting diodes OLEDs “Organic Light-Emitting Diodes”.^14^ In fact, the most striking examples of functional hybrids exhibit emission properties (solid-state dye laser, rare-earth doped materials), absorption properties (photochromic), and non linear optical (NLO) properties.^15–17^ The growth of research in nonlinear optics (NLO) is mainly related to the rapid technological advancements that have occurred in several related fields, such as ultra-fast phenomena, optical communication and optical storage devices. On the other hand, nonlinear optical crystals, which have high optical band gap, ultrafast response times and low dielectric constant, are in great demand in the optical storage devices, color display units and optical communication systems, *etc.*^18^ Moreover, compared to organic molecules, organometallic compounds showed strong absorptions in UV/V regions due to metal-to-ligand and ligand-to-metal charge transfer.^19^ On the other hand, all the properties combine to make them as a suitable candidate for multifunctional material development.^20^ In fact, several studies were performed on [TMA]_2_MeX_4_ compounds due to their variety of phase transitions. The obtained results indicate that the tetramethylammonium and the tetrachlorozincate [TMA]_2_ZnCl_4_ crystal is suitable for optoelectronic applications around room temperature. Below 296 K, this crystal can directly store the optical data and information. Moreover, the [TMA]_2_ZnCl_4_ crystal is conveniently transparent in the visible light region with low absorption coefficient in this region, which makes it suitable for anti-reflection layers of solar.^21^ However, the previous optical studies on the use of organic–inorganic hybrid materials in optoelectronic or optical application have received little attention. In fact, to use materials in the field of optoelectronics, it is necessary to know their optical constants. Additionally, optical measurements can give us a lot of information about the composition and the quality of the materials.

In fact, in the present paper, we have investigated the optical properties of [DMA]_2_MCl_4_ (M = Zn, Co) and [DMA]_2_ZnBr_4_ compounds. The results of the optical absorption were performed at room temperature. The main aim is to determine some important optical parameters, such as the bandgap energy (*E*_g_), the Urbach energy (*E*_u_), the refractive index (*n*), the oscillator energy (*E*_0_), the dispersion energy (*E*_d_), the real and imaginary parts of the complex dielectric function (*ε*) and the optical conductivity *σ*_opt_. We have also made an analysis of optical properties correlated with dielectric properties of [DMA]_2_MCl_4_ (M = Zn, Co) and [DMA]_2_ZnBr_4_ single crystals. These results were analyzed and discussed in the light of published data of some A_2_MX_4_ crystals.

## 2. Experiment

[DMA]_2_MCl_4_ (M = Zn, Co) crystals are prepared by dissolving stoichiometric amounts of water *starting materials* N(CH_3_)_2_H_2_Cl and MCl_2_ (M = Zn, Co) in water, in a molar ratio of (2 : 1) according to the reaction scheme:2[N(CH_3_)_2_H_2_]Cl + MCl_2_ → [N(CH_3_)_2_H_2_]_2_MCl_4_

After a few days of evaporation, deep blue single crystals of [DMA]_2_CoCl_4_ and white polyhedral crystals [DMA]_2_ZnCl_4_ appeared then stored in a sealed container in order to carry out the planned studies. The crystals grew as elongated prisms.

As in the case of the compound, the [DMA]_2_ZnBr_4_, ZnBr_2_ anhydrous (Sigma-Aldrich) (1 mmol) and (CH_3_)_2_NH·HCl (Sigma-Aldrich) (2 mmol) were dissolved in a hydrobromic acid aqueous solution (30 mL) and stirred after which the obtained solution had slowly been evaporated at RT for a few days. Then, the white prism-like single crystals of [DMA]_2_ ZnBr_4_ were collected, washed with a small amount of distilled water, and dried in open air.

## 3. Apparatus

The X-ray diffraction (XRD) pattern of powder was recorded at room temperature using a Philips PW 1710 diffractometer operating with copper radiation (*λK*_α_ = 1.5406 Å) in a range of Bragg's angle (5° ≤ 2*θ* ≤ 80°) in each compound. Then, the unit cell parameters were refined with the Celref 3 software^22^ by the least square method from the powder data.

Therefore, in order to study the optical properties of [DMA]_2_MCl_4_ (M = Zn, Co) and [DMA]_2_ZnBr_4_ compounds, the UV-Vis spectra were obtained by a UV-VIS spectrophotometer (Shimadzu UV-3101PC) using a source emitting wavelength radiations varying between 200 and 800 nm on a pellet of each compound. For the optical measurements, we used crystal powder pellets of 8 mm diameter and 0.8 mm thick.

The dielectric measurements were carried out on a pellet of 8 mm diameter and 1.1 mm thick obtained by means of a press of 3 tons per cm^2^ from a finely ground powder, using a SOLARTRON SI 1260 impedance bridge, which operates in the frequency range of 10^−1^ to 10^7^ Hz. Then, the temperature is regulated using a precise regulator fitted with a thermocouple placed near the sample.

## 4. Results and discussion

### 4.1. X-ray diffraction study

**Fig. 1:**
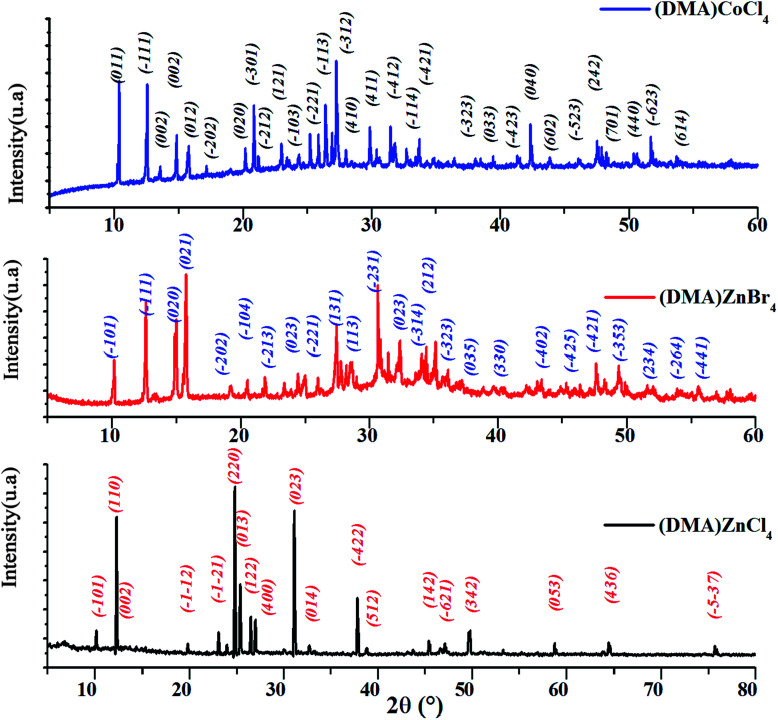
X-ray diffraction pattern of [DMA]_2_ZnCl_4_, [DMA]_2_CoCl_4_ and [DMA]_2_ZnBr_4_.

The powder X-ray diffraction pattern of [DMA]_2_MCl_4_ (M = Zn, Co) and [DMA]_2_ZnBr_4_, recorded at room temperature is shown in Fig. 1. In fact, all the peaks of [DMA]_2_ZnCl_4_ are indexed in the monoclinic system with the *P*2_1_/*n* space group while the unit cell parameters refined by the least square method are: *a* = 13.201 Å, *b* = 8.604 Å, *c* = 11.552 Å and *β* = 89.34°. These values are in good agreement with those found in the literature.^4,5^

The [DMA]_2_CoCl_4_ compound at 300 K belongs to a mono-clinic system, *P*2_1_/*n* space group, with the following unit cell dimensions: *a* = 8.5393 Å, *b* = 11.3905 Å, *c* = 13.4069 Å and *β* = 91.02°. Then, compared to the standard value, the obtained diffraction peak positions are found to be in agreement with those of the corresponding literature, confirming the phase and purity of the compound.^10^

Moreover, all the reflection peaks corresponding to 2*θ* values of [DMA]_2_ZnBr_4_ are indexed monoclinic system with the *P*2_1_/*n* space group. The obtained lattice parameters are *a* = 8.7163 Å, *b* = 11.9837 Å, *c* = 16.1951 and *β* = 121.59°. These results are in agreement with the published results.^12^

### 4.2. Optical absorption

**Fig. 2:**
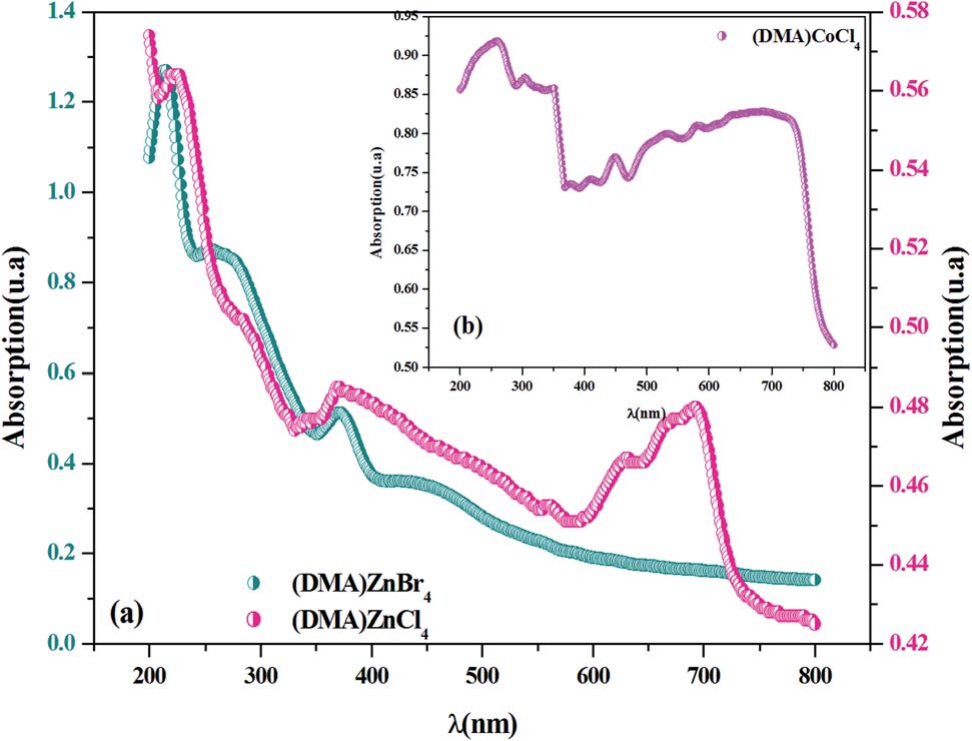
(a and b) UV-visible absorption spectra for [DMA]_2_ZnCl_4_, [DMA]_2_ZnBr_4_and [DMA]_2_CoCl_4_ at room temperature.

Fig. 2(a) shows electronic absorption spectra of [DMA]_2_ZnCl_4_, in comparison to [DMA]_2_ZnBr_4_. In fact, in the UV region, a broad absorption band corresponds to three peaks for each compound. The absorption increases sharply at 222 nm and 213 nm, which is the characteristic of the fundamental band of [DMA]_2_ZnCl_4_ and [DMA]_2_ZnBr_4_ materials, respectively. It is due to the excitation of an electron from the valence band to the conduction band, *i.e.* band-to-band transition, in the [ZnCl_4_]^2−^ and [ZnBr_4_]^2−^ tetrahedron, indicating the material gap. However, the other two bands observed at (283 nm, 370 nm) for [DMA]_2_ZnCl_4_ and at (256 nm, 371 nm) and for [DMA]_2_ZnCl_4_, are attributed to the excitonic transition. These results are quite similar to those found for other previously reported MX^4−^hybrids, such as [(C_2_H_5_)NH_3_]_2_ZnCl_4_ and [N(CH_3_)_4_]_2_ZnCl_4_.^23,24^ As for its structure, [DMA]_2_ZnCl_4_ is close to isomorphous tetramethylammonium. The observed band positions are very similar to the [TMA]_2_ZnCl_4_ spectrum.^25^ The peak around 692 nm in [DMA]_2_ZnCl_4_ spectrum can be assigned to –NH– species.

Fig. 2(b) shows the experimental UV-Vis absorption spectrum of [DMA]_2_CoCl_4_ at room temperature. As previously seen, there are eight distinct absorption peaks. The spectrum shows three absorption bands in the UV region centered at 376 nm, 304 nm and 258 nm, corresponding to the low energy tail of the band, corresponding to ligand-to-metal charge transfer (3p Cl ! 3d Co).^26^ Additionally, the absorption spectrum exhibits five peaks in the visible domain, located at 635 nm, 579 nm, 534 nm, 448 nm and 405 nm, probably assigned to internal electron transitions of the 3 d^7^ configuration of a Co^2+^ ion in [CoCl_4_]^2−^.^27–29^ The observed band positions are very similar to those found in other materials containing organic–inorganic hybrid compound previously reported in the literature.^26,30–32^ As expected from some literature data, the corresponding transitions from the initial ^4^A_2_(^4^F) state into the excited states are grouped in Table 1.^30,32^ It is also interesting to note that with respect to the mutual position of the bands, the [DMA]_2_CoCl_4_ spectrum is very close to the [TMA]CoCl_4_ spectrum.^27^ In the UV region, one can observe that the absorbance values of the Br-based compound are higher than those of the Cl-based compounds. Moreover, from absorption spectra, the crystals absorb UV radiation, which may be used as an effective UV shelter.^33^

**Table 1:** Assignment of absorption bands in spectrum of [DMA]_2_CoCl_4_

Band (nm)	Transition from ^4^A_2_(^4^F)
258	^2^T_1_(^2^F)
304	^2^A_2_, ^2^T_2_(^2^F)
376	^2^E(^2^D)
405	^2^T_2_(^2^D)
448	^2^T_1_, ^2^T_2_, ^2^E, ^2^T_1_, ^2^T_1_(^2^P^2^H)
534	^2^T_1_, ^2^A_1_, ^2^T_2_(^2^G)
579	^4^T_1_(^4^P)
635	^2^E(^2^G)

### 4.3. Direct and indirect optical band gap and Urbach energy

The band gap energy is determined using the Tauc relation:^34^

1 *αhν* = *B*(*hν* − *E*_g_)^*n*^

with *α* the optical absorbance, *hν* the photon energy, *B* a constant, then *E*_g_ the optical band gap, *n* = ½ or 2 for direct and indirect allowed transition, respectively.

**Fig. 3:**
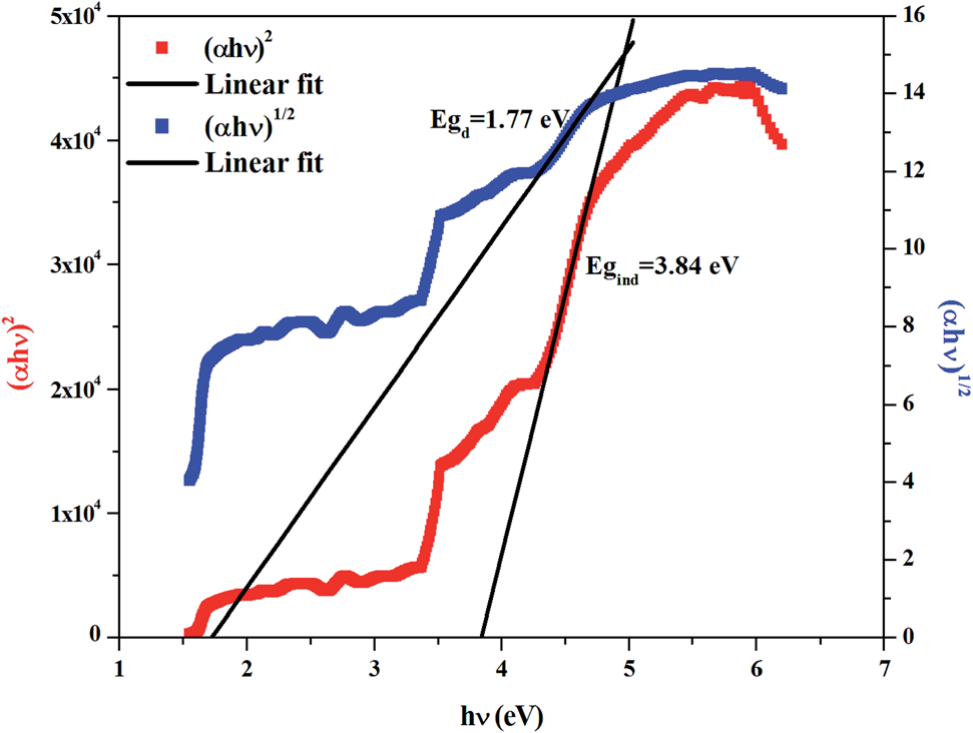
Variations of (*αhν*)^2^ and (*αhν*)^1/2^ as a function of the photon energy for the compound [DMA]_2_ZnCl_4_.

**Fig. 4:**
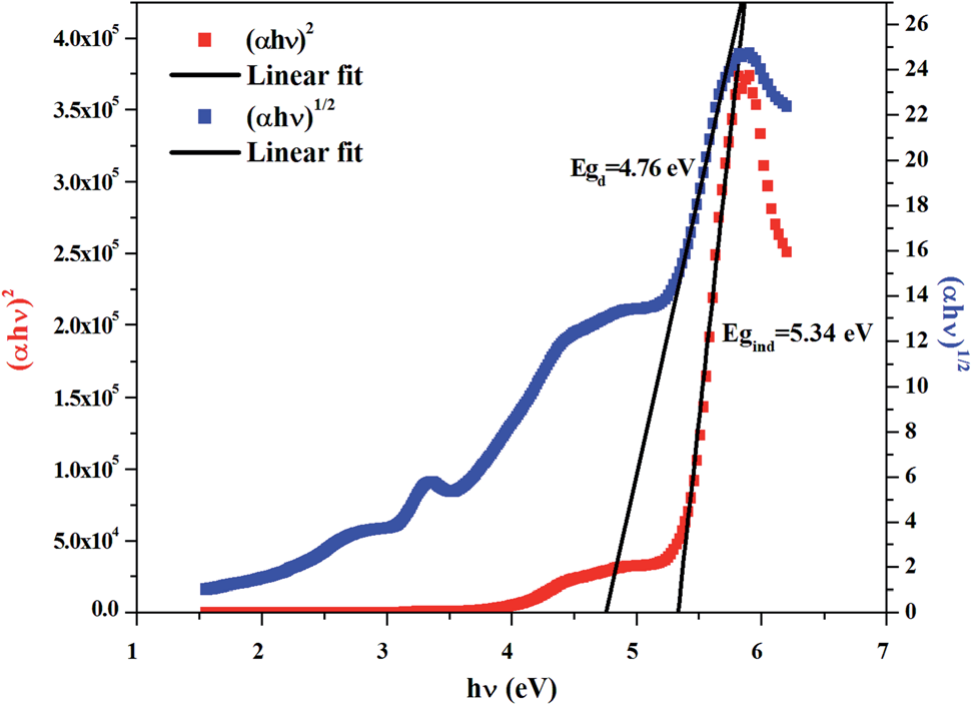
Variations of (*αhν*)^2^ and (*αhν*)^1/2^ as a function of the photon energy for the compound [DMA]_2_ZnBr_4_.

**Fig. 5:**
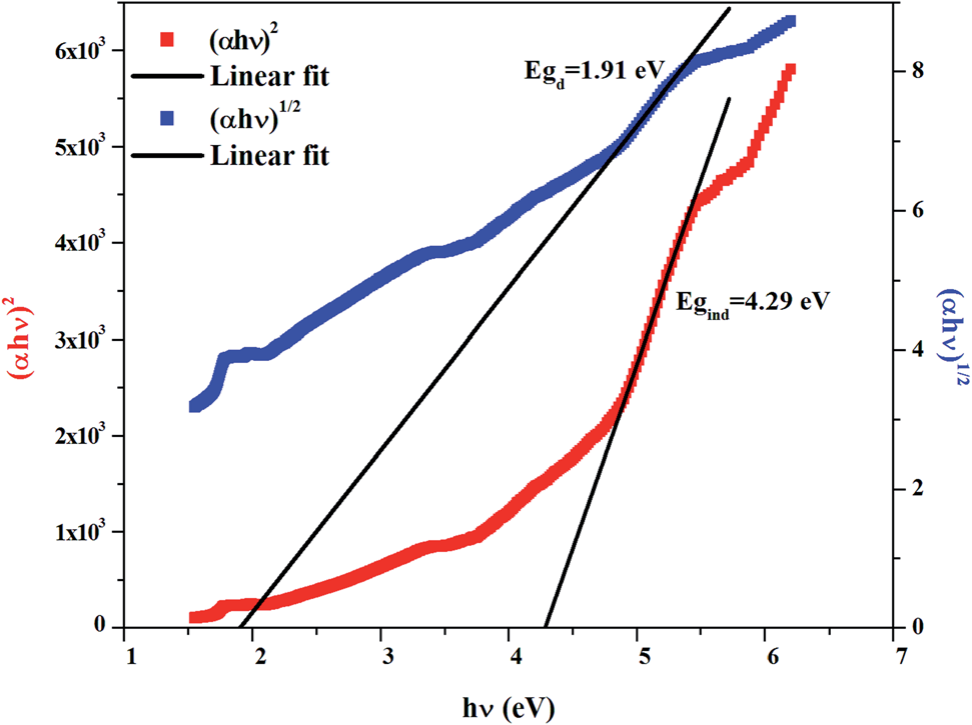
Variations of (*αhν*)^2^ and (*αhν*)^1/2^ as a function of the photon energy for the compound [DMA]_2_CoCl_4_.

The dependence of (*αhν*)^2^ and (*αhν*)^1/2^ on the photon energy is shown in Fig. 3–5 for DMA]_2_ZnCl_4_, [DMA]_2_ZnBr_4_ and [DMA]_2_CoCl_4_compounds, respectively. Then, the obtained intersection of the straight line, which is divided by the slope, is equal to the energy band gap of optical transitions.^35^ On the other hand, the optical edge or gap was inferred by linear extrapolation of the absorbance from the high slope region obtained from the spectra. It can also be noted that if erroneously the band gap energy is related directly to the absorption peaks, which appear in as-collected UV-Vis spectra, it can lead to overestimate its value with smaller discrepancies. In fact, the values of the band gap energy (*E*_g_) of the samples at room temperature are listed in Table 2. These results are similar to those obtained for other similar hybrid materials^36–40^ (Table 3). Then, the calculated *E*_g_ values for [DMA]_2_CoCl_4_ crystals are lower than those for [DMA]_2_ZnCl_4_and [DMA]_2_ZnBr_4_. The band gap energy value for [DMA]_2_CoCl_4_ suggests that the material behaves as semiconductor. However, [DMA]_2_ZnCl_4_ and [DMA]_2_ZnBr_4_ compounds can be classified as wide-gap semiconductors. On the other hand, it should be noted that *E*_g_ corresponding to [DMA]_2_ZnCl_4_ is lower than that of [DMA]_2_ZnBr_4_.

**Table 2:** Calculated values of band gaps and Urbach energy

Sample	*E*_gd_ (eV)	*E*_gi_ (eV)	*E*_u_ (eV)
[DMA]_2_ZnCl_4_	1.91	4.29	2.19
[DMA]_2_ZnBr_4_	4.76	5.34	0.36
[DMA]_2_CoCl_4_	1.77	3.84	1.81

**Table 3:** Gap energy and Urbach energy of some hybrid compounds of A_2_MX_4_ types

Sample	*E*_g_ (eV)	*E*_u_ (eV)
[N(CH_3_)_4_]_2_CoCl_4_	4.598 [ref. 36]	—
[C_8_H_10_NO]_2_CoCl_4_	2.98 [ref. 37]	1.27 [ref. 37]
[N(CH_3_)_4_]_2_ZnCl_4_	5.903 [ref. 38]	0.73 [ref. 21]
[N (CH_3_)_4_]_2_MnCl_4_	5.419 [ref. 36]	—
[(C_2_H_5_NH_3_]_2_ZnCl_4_	4.04/4.55 [ref. 39]	2.19 [ref. 39]
[C_8_H_10_NO]_2_CdCl_4_	3.17 [ref. 40]	0.622 [ref. 40]

Therefore, in order to obtain information on the disorder in a material, an Urbach tail analysis was also performed. In this context, Urbach's rule describes the broadening of the absorption edge and the formation of a band tail. Thus, Urbach tail energy (*E*_u_) is introduced to describe the width of the tails due to localized states in the absorption edge.^41^ We can access the value of *E*_u_ from the following equation:2
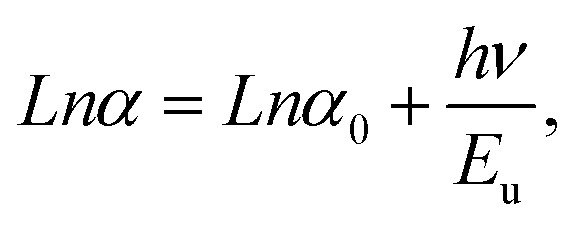
where *α*_0_ is a constant, *E*_u_ represents the Urbach energy (eV) and *hν* is the photon energy (eV).

**Fig. 6:**
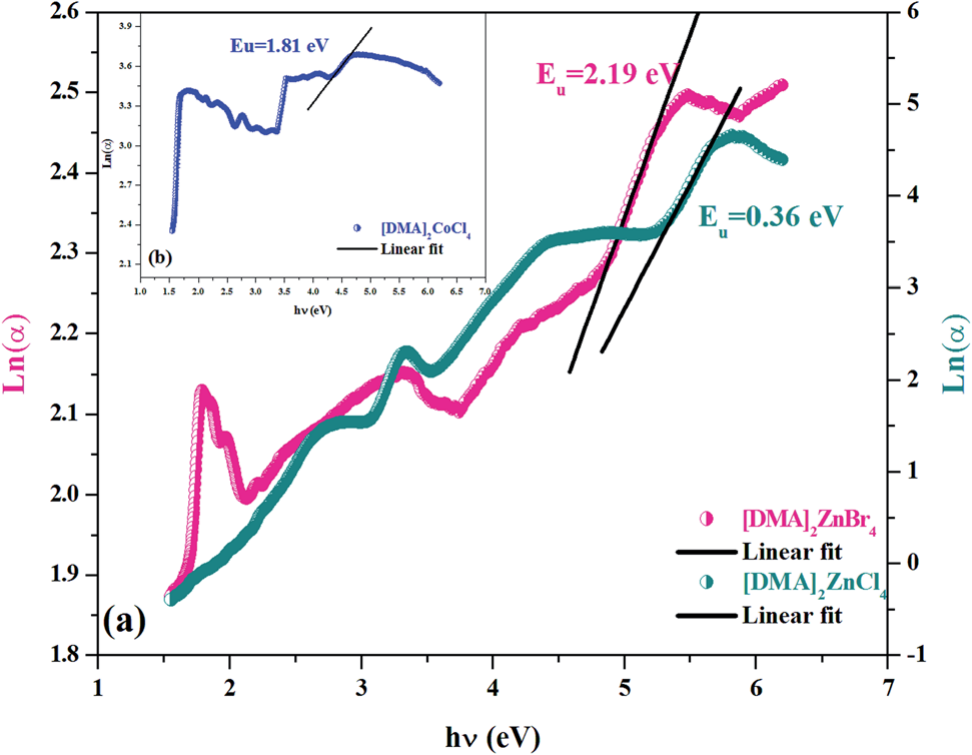
(a and b) Variation of *Ln*(*α*) as a function of (*hν*) for [DMA]_2_ZnCl_4_, [DMA]_2_ZnBr_4_ and [DMA]_2_CoCl_4_.

As seen in Fig. 6(a and b), the tail cannot accurately fit a single slope in the *Ln*(*α*) *vs.* photon energy plot. As seen from Table 2, each of the investigated [DMA]_2_MeX_4_ is characterized by its value *E*_u_. These values were similar to the band tails found in the literature (Table 3).^21,37,39,40^ It is clear that the energy *E*_u_ is higher for the Cl-based compound compared to the Br-based compounds. The large value may be because of the high degree of defect in the cell.^42^ Thus, the rate of recombination in perovskite compounds increased as *E*_u_ went up because of the rise of the density of the localized states lying deeply between the band gaps.

### 4.4. Optical constants

#### 4.4.1. Extinction coefficient

When optical energy interacts with the matter, it disperses and consequently losses occur, which are described by the extinction coefficient. Then, the absorption coefficient *α* is related to the extinction coefficient *k* by:3
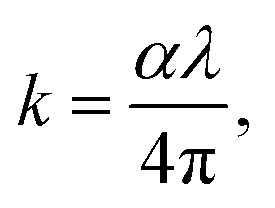
where *k* represents the imaginary part of the complex refractive index (*N* = *n* + *ik*) and *λ* is the wavelength of the incident photons, which is responsible for the attenuation of the electromagnetic wave in the medium.

**Fig. 7:**
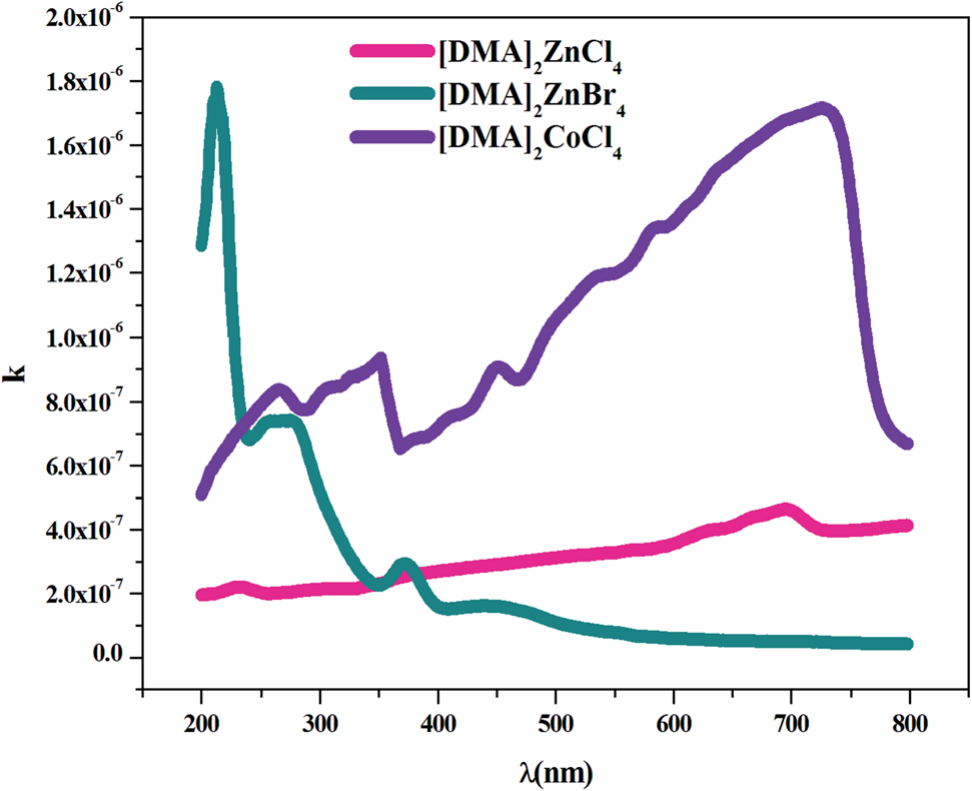
Variation of the extinction coefficient *k* as a function of the wavelength *λ* for [DMA]_2_ZnCl_4_, [DMA]_2_ZnBr_4_ and [DMA]_2_CoCl_4_.

In fact, Fig. 7 shows the plot of the extinction coefficient *k* as a function of the wavelength *λ* (nm) for [DMA]_2_ZnCl_4_, [DMA]_2_ZnBr_4_ and [DMA]_2_CoCl_4_. Moreover, the fall and rise in the extinction coefficient are due to the variation of the absorbance. In fact, low values of *k* indicate the region of transparency of each compound. On the other hand, for the compound dimethylammonium tetrachlorocobaltate, the extinction coefficient *k* shows several peaks and reaches a maximum in the visible region due to the dark blue color of the crystal. However, the two compounds [DMA]_2_ZnCl_4_ and [DMA]_2_ZnBr_4_ transmit almost most of the incident radiation due to their total transparency in the visible area. In fact, when the crystals exhibit a wide transmission range in the visible domain, they can be used for nonlinear optical applications and antireflection layers of solar thermal devices.^21^

The Br-based compound showed *k*-values lower than the Cl-based compounds in the visible light spectrum. Therefore, these adequate results justify the use of this material in photovoltaic devices.^43^

#### 4.4.2. Refractive index

The refractive index, which is the result of complex phenomena of interaction between fields and atoms of the matter, can be expressed by:4
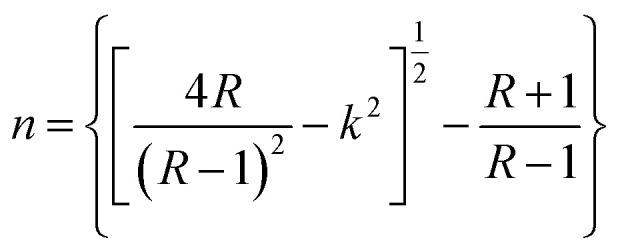
5
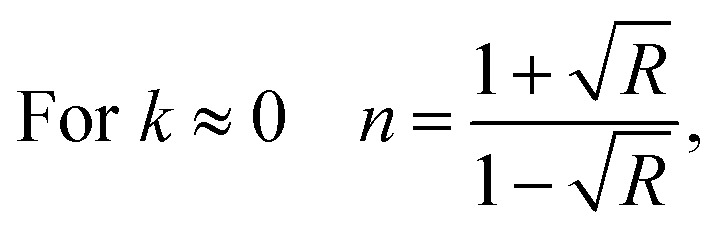
where *R* is the reflectance of the surface and *k* is the extinction coefficient. Then, the variation of the (*n*) index, as a function of the wavelength, can be described by two models; the Cauchy and the Wemple–DiDomenico models.

The Cauchy's formula of the refractive (*n*) index, as a function of the wavelength *λ*, is:^44,45^6
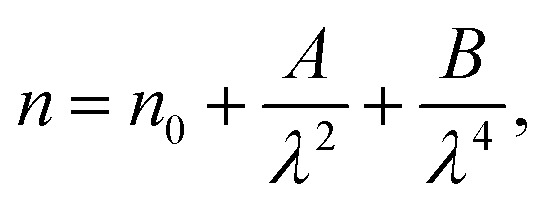
where (*n*_0_, *A* et *B*) correspond to the Cauchy's constants and λ is the wavelength of the incident photons.

**Fig. 8:**
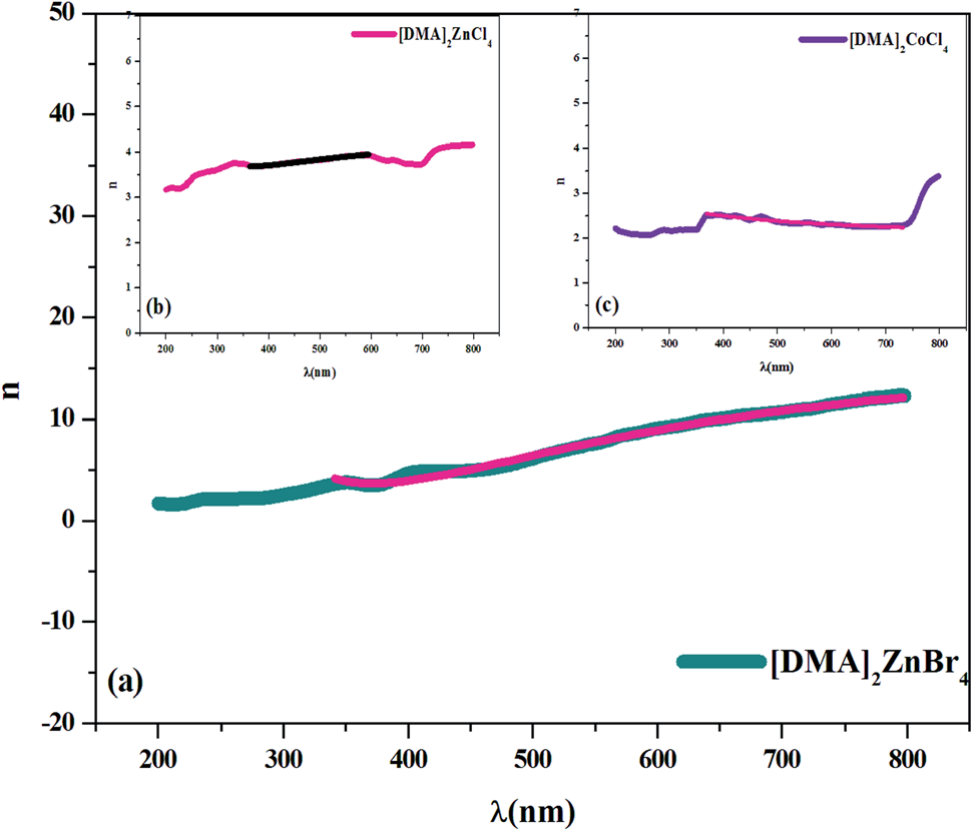
(a–c) Plot of the refractive index *versus* wavelength *λ* (nm) for [DMA]_2_ZnBr_4_, [DMA]_2_ZnCl_4_ and [DMA]_2_CoCl_4_, respectively.

In fact, Cauchy suggests that the refractive index depends primarily on the material and the wavelength. The dependence of the refractive index on the *λ* (nm) wavelength for [DMA]_2_ZnCl_4_, [DMA]_2_ZnBr_4_ and [DMA]_2_CoCl_4_ is also shown in Fig. 8(a–c), respectively. The increase of the refractive index is due to the increase of the reflectance. The values of *n*_0_, *A* and *B* are grouped in Table 4. These estimated values are close to the ones reported by.^41,46^ It is clear that *n*_0_ is very large for the compound [DMA]_2_ZnBr_4_ compared to the [DMA]_2_ZnCl_4_ and [DMA]_2_CoCl_4_ compounds. We also notice that for the Cl-based compounds, *n* is almost constant, that is to say that the variation of *n* depends very little on *λ* (*A* and *B* are very weak); however, in the compound [DMA]_2_ZnBr_4_, *n* is very sensitive to the variation of the wavelength (*A* and *B* are greater than for the other two compounds).

**Table 4:** Calculated values of Cauchy's parameters *n*_0_, *A* and *B*

Sample	*n*_0_	*A* (μm^2^)	*B* (μm^4^)
[DMA]_2_ZnCl_4_	4.32	−0.16	1.04 × 10^−2^
[DMA]_2_ZnBr_4_	17.55	−3.84	0.266
[DMA]_2_CoCl_4_	2.08	0.08	3.51 × 10^−3^

**Fig. 9:**
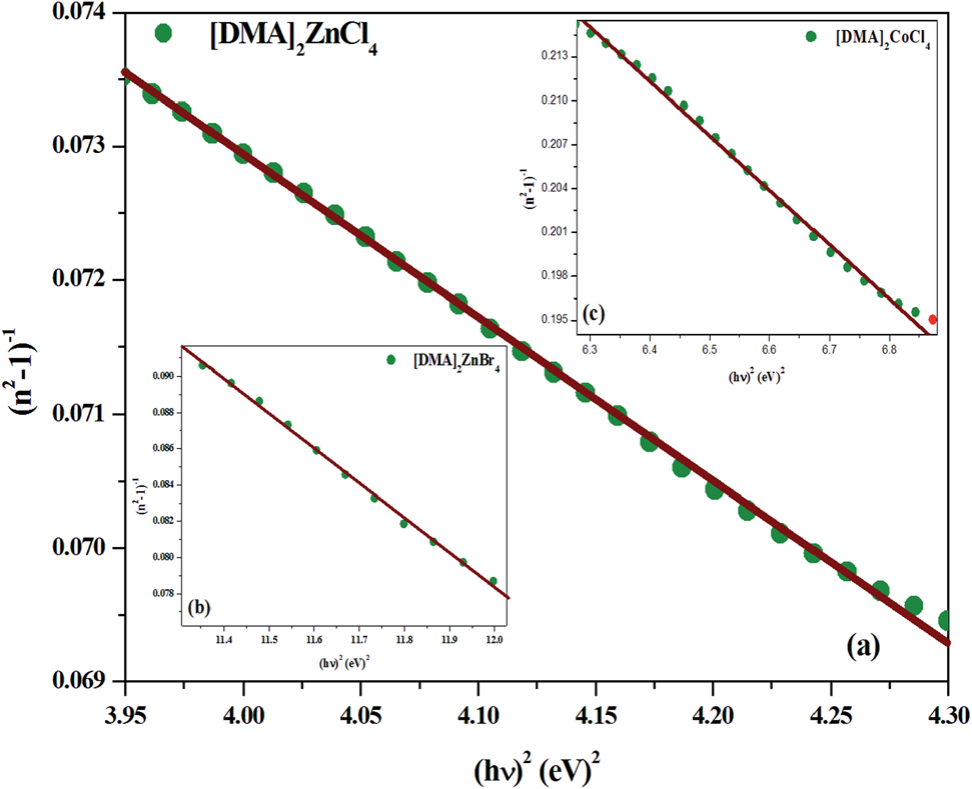
(a–c) Plots of (*n*^2^ − 1)^−1^*versus* (*hν*)^2^ of [DMA]_2_ZnCl_4_, [DMA]_2_ZnBr_4_ and [DMA]_2_CoCl_4_, respectively.

The concept of the single oscillator suggested by Wemple–DiDomenico describes the evolution of the refractive *n* index by the following equation:^47–49^7
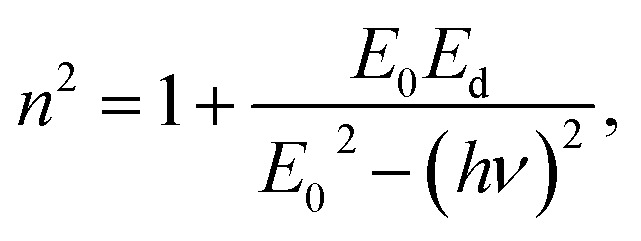
where *E*_0_ is the single-oscillator energy and *E*_d_ the dispersion energy. In fact, *E*_0_ corresponds to the energetic distance between the valence and conduction bands and *hν* is the photon energy (eV). Then, the parameter *E*_d_, which is a measure of the average strength of the inter-band optical transitions, does not significantly depend on the band gap. Moreover, parameters *E*_0_ and *E*_d_ for [DMA]_2_ZnCl_4_, [DMA]_2_ZnBr_4_ and [DMA]_2_CoCl_4_ hybrids were calculated from the slope of the linear portion of the plot (*n*^2^ − 1)^−1^*versus* (*hν*)^2^ as illustrated in Fig. 9(a–c), respectively. Then, the values obtained for the oscillating parameters are gathered in Table 5.

**Table 5:** Calculated values of *E*_d_, *E*_0_, *n*_∞_, *λ*_0_ and *S*_0_

Sample	*E*_0_ (eV)	*E*_d_ (eV)	*n*_∞_	*λ*_0_ (nm)	*S*_0_ (nm^−2^) × 10^−5^
[DMA]_2_ZnCl_4_	9.98	8.21	1	316	8.20
[DMA]_2_ZnBr_4_	4	12.98	5.29	356	3.38
[DMA]_2_CoCl_4_	3.47	7741	2.67	309	1.75

Therefore, the previous equation can be written as follows:8
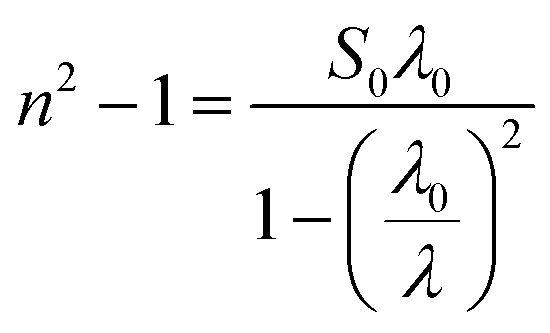
where *λ*_0_ is the average oscillator wavelength, *S*_0_ corresponds to the oscillator length strength and *λ* is the wavelength of the incident light. Therefore, in order to determine the values of the long wavelength refractive (*n*_∞_), index average oscillator wavelength *λ*_0_ and the average oscillator strength (*S*_0_) for the samples, we can use the following relationship:9
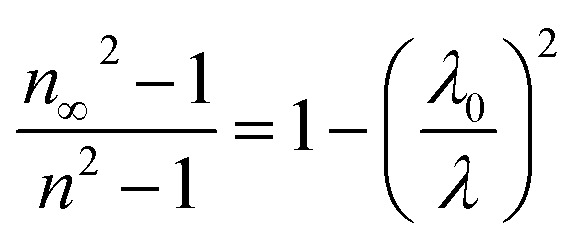
and

10 *n*_∞_^2^ = 1 + *S*_0_*λ*_0_^2^

**Fig. 10:**
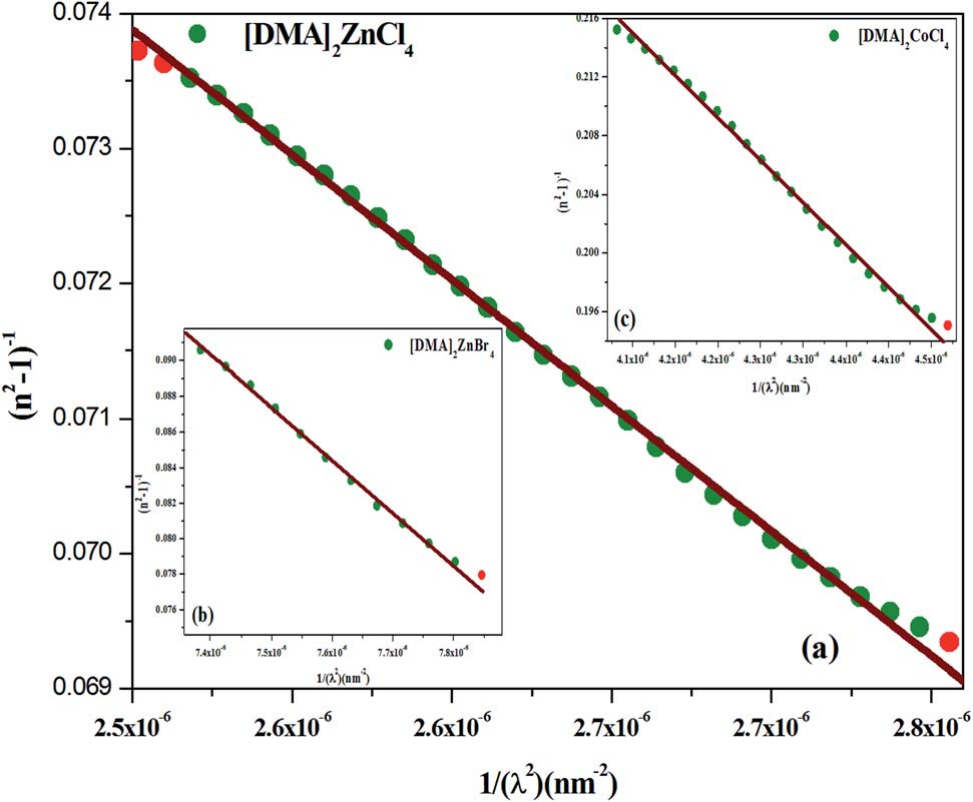
(a–c) Plots of (n^2^-1)^−1^*versus* 1/*λ*^2^ of [DMA]_2_ZnCl_4_, [DMA]_2_ZnBr_4_ and [DMA]_2_CoCl_4_, respectively.

The plot of (*n*^2^ − 1)^−1^, as a function of (1/*λ*^2^), gives a linear part (Fig. 10(a–c)) for [DMA]_2_ZnCl_4_, [DMA]_2_ZnBr_4_, [DMA]_2_CoCl_4_, successively. In fact, Table 5 shows the values of *n*_∞_, *λ*_0_ and *S*_0_.

On the other hand, for the development of optical applications of the optical material under study, determining the moments of the spectrum is very necessary. Therefore, the *M*_−1_ and *M*_−3_ moments can be determined by the following equations:11
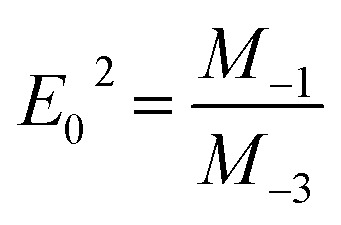
And,12
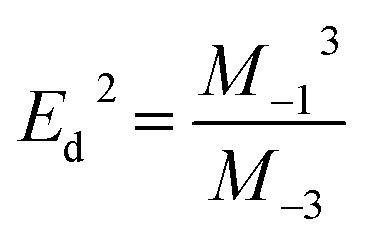
The *M*_−1_ and *M*_−3_ values of for [DMA]_2_ZnCl_4_, [DMA]_2_ZnBr_4_ and [DMA]_2_CoCl_4_ compound are summarized in Table 6. Then, the obtained *M*^−1^ and *M*^−3^ are slightly elevated for the compound [DMA]_2_ZnBr_4_. These moments determine the average bond strength. Eqn (11) and (12) indicates a single-oscillator approximation to the dielectric response of these materials. The optical moments are related to the macroscopic quantities, like the effective dielectric constant, the effective number of valence electrons in the investigated material.^50^

**Table 6:** Calculated values of *M*_−1_ and *M*_−3_

Sample	*M*_−1_ ((eV)^−2^)	*M*_−3_ ((eV)^−2^)
[DMA]_2_ZnCl_4_	0.82	0.00825
[DMA]_2_ZnBr_4_	3.23	0.20096
[DMA]_2_CoCl_4_	2.22	0.18373

#### 4.4.3. Dielectric constant

Several theoretical models have been developed to study the dielectric constants for material applications in optoelectronic devices, which characterize the optical properties of a solid material. In fact, the real part (*ε*_r_) of the dielectric constant shows how much it will slow down the speed of light in the material, whereas the imaginary part (*ε*_i_) represents the absorption of the associated radiation by free carriers. The complex dielectric constant *ε* is related to the refractive index (*n*) and the extinction coefficient (*k*) as:^49^

**Fig. 11:**
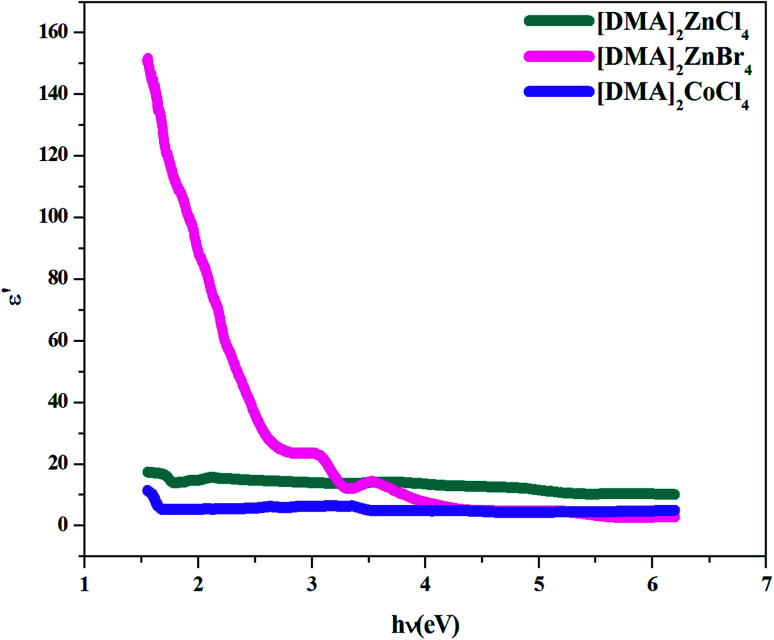
Real part *ε*′ of the dielectric permittivity *versus* (*hν*) for [DMA]_2_ZnCl_4_, [DMA]_2_ZnBr_4_ and [DMA]_2_CoCl_4_.

**Fig. 12:**
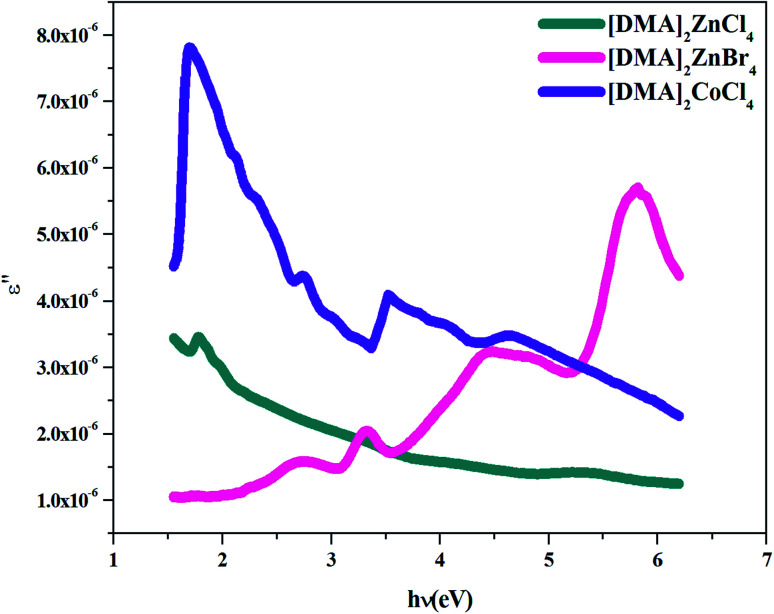
Imaginary part *ε*′ of the dielectric permittivity *versus* (*hν*) for [DMA]_2_ZnCl_4_, [DMA]_2_ZnBr_4_ and [DMA]_2_CoCl_4_.

13 *ε* = *ε*_r_ + *iε*_i_,

14 *ε*_r_ = *n*^2^ − *k*^2^,

15 *ε*_i_ = 2*nk*,

Additionally, studies of the real and imaginary parts of the dielectric constant give us the information about the loss factor, which is the ratio of the imaginary part to the real part of the dielectric constant. Then, the variation of the real and imaginary part of the dielectric constant with the incident photon energy for the [DMA]_2_ZnCl_4_, [DMA]_2_ZnBr_4_, [DMA]_2_CoCl_4_ sample were determined and shown in Fig. 11 and 12.

On the other hand, the extinction coefficient has a negligible contribution (in the order of 10^−6^) whereas the refractive index has a comparatively very high value. An increase of the real part was observed with the increase of the photon energy. Consequently, the real part of the dielectric constant depends only on the refractive index by eqn (7). For the [DMA]_2_ZnBr_4_ compound, the calculated real dielectric constant values are very high (10–155) at the particular interest of the visible region. The imaginary part of the dielectric constant depends on the extinction coefficient by eqn (8). As can be seen from Fig. 12, different shapes of the curves for the imaginary part of the dielectric constant have been observed. Therefore, the imaginary part confirms the free carrier contribution to the absorption. We notice that the imaginary part of the complex permittivity (in the order of 10^−6^) is very low compared to the real part.

#### 4.4.4. Optical conductivity

**Fig. 13:**
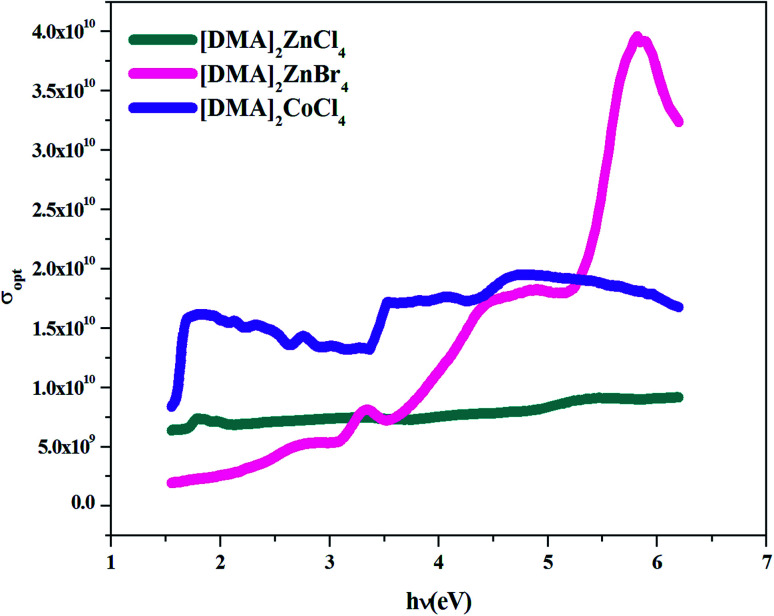
*σ*_opt_ *versus* (*hν*) for [DMA]_2_ZnCl_4_, [DMA]_2_ZnBr_4_and [DMA]_2_CoCl_4_.

Optical conductivity is a significant tool for studying the electronic states in materials. In general, if an external electric field is applied, a redistribution of charges occurs and currents are induced. Fig. 13 shows the variation of optical conductivity with the incident photon energy. Optical conductivity was determined using the relation:^51,52^

16 *σ* = *αnc*/4π,

where *c* is the velocity of light and *α* is the absorption coefficient.

The increased optical conductivity is due to the high absorbance of the sample in that region. The optical conductance and band gap indicated that [DMA]_2_ZnCl_4_ and [DMA]_2_ZnBr_4_ have high transmittance compared to [DMA]_2_CoCl_4_ within the visible range.

The maximum value of conductivity for the compound [DMA]_2_ZnBr_4_ (4 × 10^10^ s^−1^) is greater than that of the two compounds [DMA]_2_ZnCl_4_ (5.5 × 10^9^ s^−1^) and [DMA]_2_CoCl_4_ (1.5 × 10^10^ s^−1^). Besides that, these high values of the optical conductivity of [DMA]_2_ZnBr_4_ pointed out to the superiority of the material for such photovoltaic applications.^53^

### 4.5. Dielectric relaxation

The dielectric properties are correlated with the electro-optic properties of the prepared crystals. Therefore, the dielectric relaxation studies are important to provide the conduction processes (*T*_g_*δ* = *ε′′*/*ε*′ ≫ 10), since it enables to determine the origin of the dielectric losses.^54^ Then, in order to obtain more information about [DMA]_2_ZnBr_4_, [DMA]_2_CoCl_4_ and [DMA]_2_ZnCl_4_ dynamic compounds, we measured the impedance spectra of the sample at room temperature from which we derived the frequency-dependent dielectric constant (*ε*′) and the loss tangent (*T*_g_(*δ*)) in the frequency range of 10^−1^ to 10^7^ Hz.

**Fig. 14:**
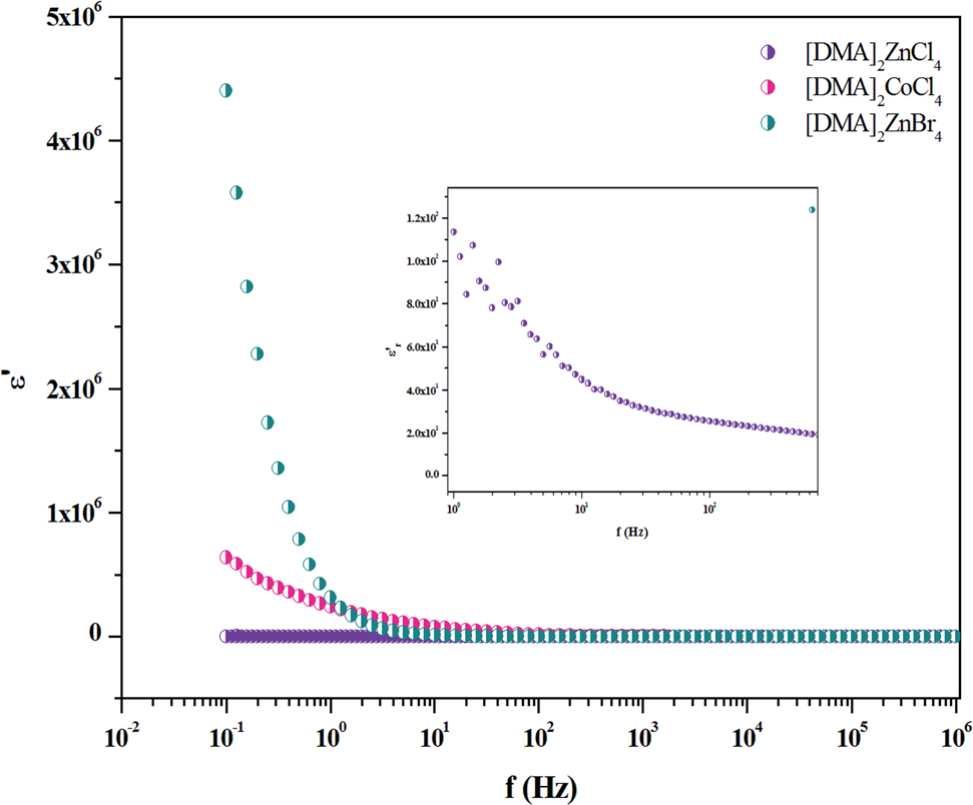
Variation of *ε*′ *versus* frequency for [DMA]_2_ZnCl_4_, [DMA]_2_ZnBr_4_ and [DMA]_2_CoCl_4_ matrix at room temperature.

The frequency dependence of the dielectric constant is depicted in Fig. 14. It is clearly that *ε*′ shows dispersions at low frequencies and gets almost saturated at higher frequencies. Such dispersions in both components of the complex dielectric constant are referred to as low frequency dielectric dispersion (LEDD) and are associated with the space charge accumulation effect and/or conducting ion motion. The observed sharp decrease of *ε*′ as frequency increases is mainly due to the space charges contributing to the high permittivity in the materials. The magnitude of the dielectric constant depends on the degree of polarization and the charge displacement in crystal. It can be observed that *ε*′ approaches a limiting constant value 
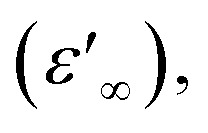
 at high frequencies, which can be attributed to the absence of space charge polarization near the grain boundary interface. Moreover, with the increase of frequency, *ε*′ becomes almost parallel. In fact, the most striking feature is that there are no relaxation peaks observed in the frequency range employed in this study. The dielectric constant decreases from a low frequency value of about ≈155 for [DMA]_2_ZnCl_4_, ≈2 × 10^5^ for [DMA]_2_CoCl_4_ and ≈4 × 10^6^ for [DMA]_2_ZnBr_4_ to the high frequency value in the order of ten. Then, the high dielectric constant of [DMA]_2_ZnBr_4_, implies that this compound could be a candidate in energetic devices. Indeed, materials of a large dielectric constant can be used as a dielectric gate or an active channel in FET devices.^55^

**Fig. 15:**
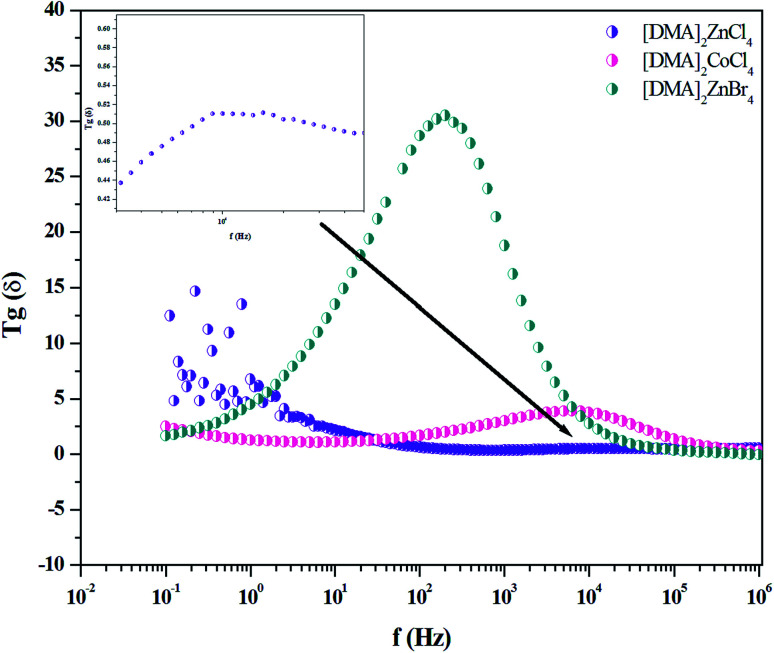
Variation of *T*_g_(*δ*) *versus* frequency for [DMA]_2_ZnCl_4_, [DMA]_2_ZnBr_4_and [DMA]_2_CoCl_4_ matrix at room temperature.

The analysis of Fig. 15 describing *T*_g_(*δ*) revealed one relaxation peak for each hybrid. This peak is noticed at the frequencies of 10^2^, 7 × 10^3^and 10^4^ Hz for [DMA]_2_ZnBr_4_, [DMA]_2_CoCl_4_ and [DMA]_2_ZnCl_4_, respectively. Considering the low frequency and the broad peak feature, this relaxation process could be a Maxwell–Wagner type interfacial polarization relaxation, possibly attributed to grain boundary effects or blocking at the contacts. From the graph, it is clear that the for [DMA]_2_ZnBr_4_, [DMA]_2_CoCl_4_ and [DMA]_2_ZnCl_4,_ the crystals exhibit very low dielectric loss at high frequencies, which can be a potential candidate for nonlinear optics NLO applications.^56^

## 5. Conclusions

In the present study, the powder XRD study of [DMA]_2_ZnCl_4_, [DMA]_2_ZnBr_4_ and [DMA]_2_CoCl_4_ crystals confirmed the lattice parameter values. The optical properties, which are of scientific importance, were investigated by optical spectroscopy (UV-visible). The obtained results show a significant difference in the absorption spectra among [DMA]_2_ZnCl_4_, [DMA]_2_ZnBr_4_ and [DMA]_2_CoCl_4_ sample. The Tauc model was used to determine the optical band gap energy values. In fact, the values of (*E*_gi_, *E*_gd_) are (1.91, 4.29) eV for [DMA]_2_ZnCl_4_, (4.76, 5.34) eV for [DMA]_2_ZnBr_4_ and (1.77, 3.84) eV for [DMA]_2_CoCl_4_ are in conformity with the reported values for other similar hybrid materials, specially [TMA]_2_MeCl_4_ (Me = Co, Zn, …) compounds. It can be observed that the band gap of all the samples lie in the range of semiconductors. Then, the observed variation in optical parameters, such as the refractive index (*n*), the extinction coefficient (*k*), the optical conductivity (*σ*_opt_), and the dielectric constants (ε*) have been evaluated and show sufficient agreement with the values reported for some A_2_BX_4_ family single crystals. The refractive index values have been fitted to the single oscillator Wemple–DiDomenico (WDD) model. The single oscillator energy (*E*_0_), the dispersion energy (*E*_d_), the static refractive index *n*_0_, the moments of the optical dispersion spectra *M*_−1_ and *M*_−3_, the static refractive index *n*_∞_ and the oscillator strength *S*_0_ were estimated at room temperature, which showed sufficient agreement with the values reported for the A_2_BX_4_ family. On the other hand, the electrical properties of the high dielectric constant at low frequencies, suggest that [DMA]_2_ZnBr_4_ could be used in the energy devices. In fact, the low dielectric constant and the low dielectric loss at high frequencies, suggest that the [DMA]_2_ZnCl_4_, [DMA]_2_ZnBr_4_ and [DMA]_2_CoCl_4_ crystalline compounds could be used in nonlinear optoelectronic devices.

## Conflicts of interest

There are no conflicts to declare.

## Supplementary Material
